# Calcium Sulfate with Stearic Acid as an Encouraging Carrier for Reindeer Bone Protein Extract

**DOI:** 10.3390/ma4071321

**Published:** 2011-07-21

**Authors:** Hanna Tölli, Elli Birr, Kenneth Sandström, Timo Jämsä, Pekka Jalovaara

**Affiliations:** 1 Division of Orthopaedic and Trauma Surgery, Department of Surgery, Institute of Clinical Medicine, Oulu University Hospital, FIN-90014 University of Oulu, Finland; E-Mail: pekka.jalovaara@oulu.fi; 2 Department of Medical Technology, Institute of Biomedicine, University of Oulu, FIN-90014 University of Oulu, Finland; E-Mail: timo.jamsa@oulu.fi; 3 BBS—Bioactive Bone Substitutes Ltd, FIN-90220 Oulu, Finland; E-Mails: elli.birr@bbs-artebone.fi (E.B.); kenneth.sandstrom@bbs-artebone.fi (K.S.); 4 Department of Diagnostic Radiology, Institute of Diagnostics, Oulu University Hospital, FIN-90014 University of Oulu, Finland

**Keywords:** bone protein extract, bone trauma, calcium salt, carrier, growth factor

## Abstract

Various bone proteins and growth factors in specific concentrations are required for bone formation. If the body cannot produce sufficient quantities of these factors, bone trauma can be healed with an implant that includes the required factors in a carrier. This study was designed to evaluate various calcium salt candidates that can be used as carrier with reindeer bone protein extract to induce ectopic bone formation in the muscle pouch model of mouse. The bone protein extract was either impregnated into the disc form of carrier or mixed with carrier powder before implantation. The radiographic analysis indicated increased bone formation in all of the active groups containing the bone protein extract compared to the controls within 21 days follow-up. The highest bone formation was seen in the group with calcium sulfate with stearic acid where new bone and calcified cartilage were clearly visible. The greatest bone formation occurred in the groups that had bone protein extract readily available. This indicates that the bone forming factors in sufficient concentrations are required at the early stage of bone formation. The calcium sulfate with stearic acid was the most suitable and effective carrier for reindeer bone protein extract.

## 1. Introduction

Native bone contains growth and differentiation factors and signaling molecules, such as bone morphogenetic proteins (BMPs) that are important for bone and cartilage regeneration [1,2]. These factors and molecules and their specific concentrations are required during the different phases of the entire fracture healing process [2]. Thus, as a treatment of bone fracture, added bone protein extract requires a suitable delivery system, or carrier, to prevent migration from the site of application with a gradual release that results in new bone formation. 

An optimal carrier matrix must fulfill several criteria. The matrix should be biocompatible, bioabsorbable, malleable, and sterilizable [3]. Inorganic materials fulfill these requirements because most of them are structurally strong, immunologically inert, highly osteoconductive and variably biodegradable [3,4]. Calcium salts, as inorganic materials, have been used for years in different variations because the composition of this material is close to that of natural bone composition [4,5,6]. 

Tricalcium phosphate (TCP) has been shown to be a useful carrier for recombinant human BMPs (rhBMPs) and demineralized bone matrix (DBM) [7,8,9]. TCP has many positive features for use in *in vivo* implants, such as resorption rates closely matching the course of normal cancellous bone remodeling and that it can bond directly to bone and has a primarily osteoconductive nature. TCP is also more soluble than hydroxyapatite (HAP) [10]. HAP is relatively osteoconductive and has high protein-binding capacity. The continuous structure of the HAP design provides a flexibility to achieve high porosity and high surface area, which makes HAP a good candidate for scaffolds [11]. However, HAP is often combined with TCP to form a more resorbable and porous carrier with a greater degree of bone formation. This combination of calcium phosphates has also been used as a carrier for rhBMPs and DBM [5,12]. Calcium sulfate has been researched as a bone void filler for over one hundred years and has many functions as part of a bone graft composite. The calcium sulfate acts as a binder to improve the total bone contact and the volume surrounding the implant [6,13]. Pore size is important for bone ingrowth, and increasing the pore size improves the bone healing effects of inorganic materials, such as calcium sulfates [14]. Calcium sulfate has been used as a carrier for DBM for a number of years, and in clinical studies it has shown excellent biocompatibility [6,13,15].

A mixture of BMPs, growth factors and other bone proteins have been extracted from the bone materials of a variety of animal species, humans and bone tumors. Previous works have demonstrated that reindeer bone protein extract is an effective stimulant for new bone formation in a muscle pouch mouse model [16,17,18]. Furthermore, the good healing capacity of the reindeer bone extract in a segmental bone defect was previously demonstrated in the rabbit and rat [19,20,21]. The ability of reindeer bone extract to heal various bone traumas is better than that of other extracts, for example, bovine or ostrich extract, which has been explained by the fact that reindeers renew their antlers annually [15,22,23]. Reindeer bone protein extract is similar in composition, method of manufacture, and intended use and application to other animal-derived bone tissue extracts. The comparable products are Colloss® and Colloss® E, which are demineralized bone extracts created from bovine and equine bone, and human demineralized bone matrix (DBM) products, such as Osteoset^®^ DBM Pellets [8,9].

This study was designed to evaluate the inorganic scaffolding components that can be used as carrier of reindeer bone extract in a mouse muscle pouch model of induced ectopic bone formation. Histological and radiographic assessments were used to determine implant responses and the potential bone formation 21 days following implantation.

## 2. Materials and Methods

### 2.1. Bone Protein Extract

The bone protein extract was extracted and purified from the diaphyseal bone of the reindeer as described previously [16]. The obtained bone protein extract was freeze-dried at −20 °C using excipients (surfactant (Polysorbat 20, Fluka, Sigma-Aldrich), lyoprotectant (D-(+)-Trehalose Dihydrate, Fluka, Sigma-Aldrich), bulking agent (Glycine, Riedel-de Haën, Sigma-Aldrich) and buffer (D-Mannitol, Fluka, Sigma-Aldrich)). 

**Figure 1:**
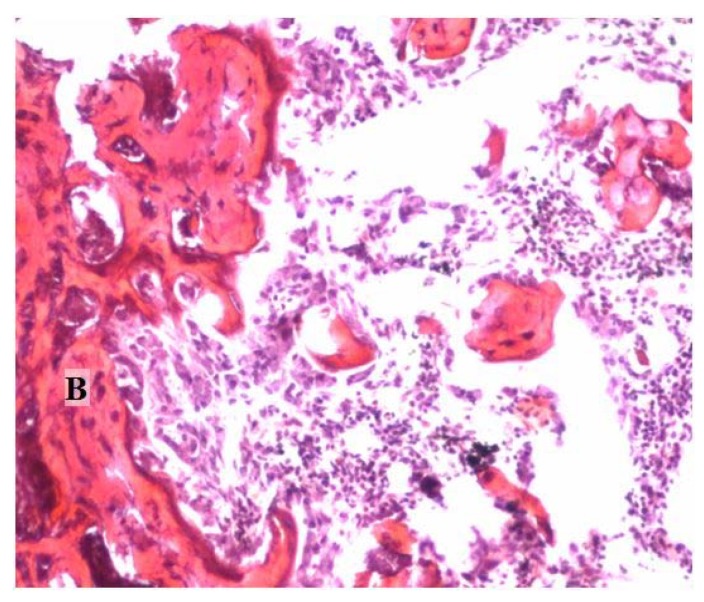
The histological examination shows the bioactivity and the new bone formation for a 3 mg dose of reindeer bone protein extract in a gelatin capsule in the mouse pouch model. B = bone. (Original magnification 10×).

The protein profile and the bioactivity of the dry bone protein extract were evaluated using the SDS-page [22] and the mouse muscle pouch model study (Figure 1).

### 2.2. Scaffold Materials and Study Groups

The used scaffold materials and study groups were (a) Porous discs that were 5 mm × 3 mm (Berkeley Advanced Biomaterials Inc, USA) with a composition of 30% hydroxyapatite (HAP), 60% tricalcium phosphate (TCP), and 10% calcium sulfate (CS); (b) Cem-Ostetic porous discs (Berkeley Advanced Biomaterial Inc., USA) that were 5 mm × 3 mm with a composition of 90% TCP and 10% CS; (c) Cem-Ostetic^®^ (Berkeley Advanced Biomaterial Inc., USA) powder for putty; (d) CS hemihydrate (97%, Sigma-Aldrich) powder for putty; (e) Non-porous discs that were 5 mm × 3 mm (Berkeley Advanced Biomaterials Inc, USA) with a composition of 60% HAP, 30% TCP and 10% CS; and (f) CS dihydrate granules with stearic acid with a composition of stearic acid 50, a mixture of fatty acids that consisted mainly of stearic acid and 40–60% palmitic acid (Fluka, Sigma-Aldrich).

### 2.3. Sample Preparation

**Figure 2:**
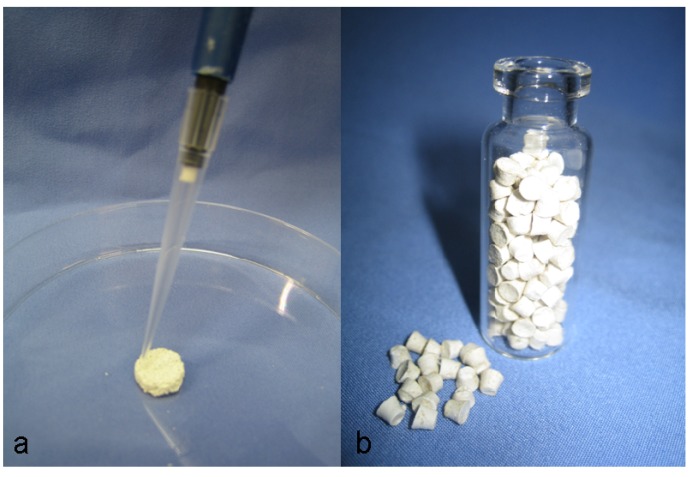
The reindeer bone protein extract was impregnated into to the carrier disc (**a**), or the protein extract together with carrier material was molded to the form of a disc (**b**).

The lyophilized reindeer bone extract (3 mg, BBS—Bioactive Bone Substitutes Ltd, Finland) was reconstituted in 0.9% physiologic saline solution (Natriumchlorid, Fagron, Tamro, Finland) and impregnated into the porous discs (a,b) and the non-porous disc (e), or mixed with the Cem-Ostetic powder (c) and CS hemihydrate (d) to form a molded disc, or dry mixed with the CS dihydrate granules and stearic acid (f) to form a compressed disc (Figure 2).

The right leg was used as a control containing the respective carrier and the excipients but excluding the bone extract.

### 2.4. Animals

A total of 48 mice of the strain BALB/c were used. Animals were supplied from the Laboratory Animal Centre, University of Oulu. Animals were 7–12 weeks of age at the time of the procedure. The study outline included 6 groups with 8 animals per group. 

One mouse from group b died on the day of the procedure without any obvious cause. One mouse from group c died on the day of the procedure due to breathing problems. Furthermore, two mice from group d and one mouse from group f were sacrificed two days after the procedure because they had issues walking. Thus, 43 mice survived until the end of the study.

### 2.5. Surgical Procedure

Surgery was performed under general anesthesia with a blend of fentanylcitrate (80 µg/kg)-fluanisone (2.5 mg/kg) (Hypnorm^®^, Janssen Pharmaceutica, Inc., Beerse, Belgium) and midazolam (1.25 mg/kg) (Dormicum^®^, Roche, Basel, Switzerland). Both legs were cleaned, and the eyes of the animals were treated with eye gel to prevent drying. The mouse was placed on a thermal mattress during the procedure. Transverse skin incisions were made near the spine at the site of the femur. Then, implants were introduced into both thigh muscle pouches in the bilateral hind legs. After the implantation, the muscles were closed with two sutures, and the skin was closed with one suture.

The pain medication post-operation consisted of buprenorphine (Temgesic^®^, Reckitt & Colman Pharmaceuticals, Inc, Richmond, England) at a dose of 0.01–0.05 mg/kg subcutaneously. The animals were allowed full activity in their cages postoperatively. All animals were euthanized 21 days after the procedure, and the hind legs were harvested. 

The study protocol was approved by the institutional animal experiment and ethical committee.

### 2.6. Radiographic Evaluation of Bone Formation

Radiographic evaluation (20 kV, 8.00 mAs, 0.32 s/exp, Mamex dc® ami, Orion Ltd., Soredex) was used to evaluate the formation of new bone and the resorption of the implant. New bone formation and resorption of the implant were evaluated by measuring the opalescent area in mm^2^ (Osiris 4.19 Digital Imaging Unit, Geneva, software). 

### 2.7. Histological Examination

Two samples from each group were prepared for histology. The specimens were fixed in 10% neutral-buffered formalin, decalcified in EDTA-formalin-solution (pH 7), processed in a tissue processor, and finally embedded in paraffin. Next, 4.5 μm-thick slices were prepared using a microtome and stained with hematoxylin-eosin. The quality of new bone and the inflammatory response on the defect site were evaluated by the histological analysis using light microscopy (Nikon Eclipse, E200, Japan).

### 2.8. Statistical Analysis

Statistical analysis was performed using the SPSS for Windows statistical package (SPSS Inc., version 15.0). The non-parametric Kruskal-Wallis test was used to evaluate the statistical differences between the groups. The Mann-Whitney test was used for pairwise comparison between the active and control groups. Values of *p* < 0.05 were considered statistically significant. The results of the radiographic assessment are given as the mean and standard deviations. The differences between the active implants and the controls are shown as percent values. 

## 3. Results

**Figure 3:**
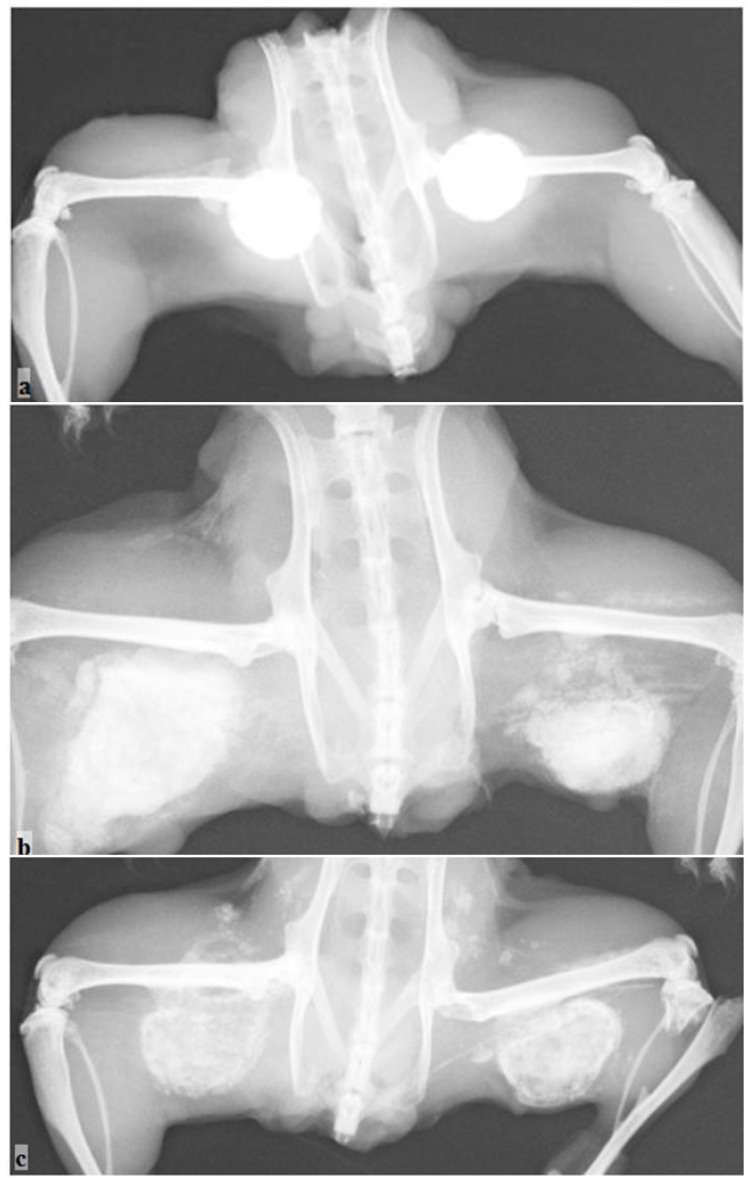
Radiographic evaluation of the new bone formation in the mouse pouch model using different carriers with the reindeer bone protein extract. The control without the bone protein extract is located on the right side, and the active implant on the left side: (**a**) HAP/TCP/CS 30:60:10; (**b**) CS hemihydrate; and (**c**) CS dihydrate + stearic acid.

**Figure 4:**
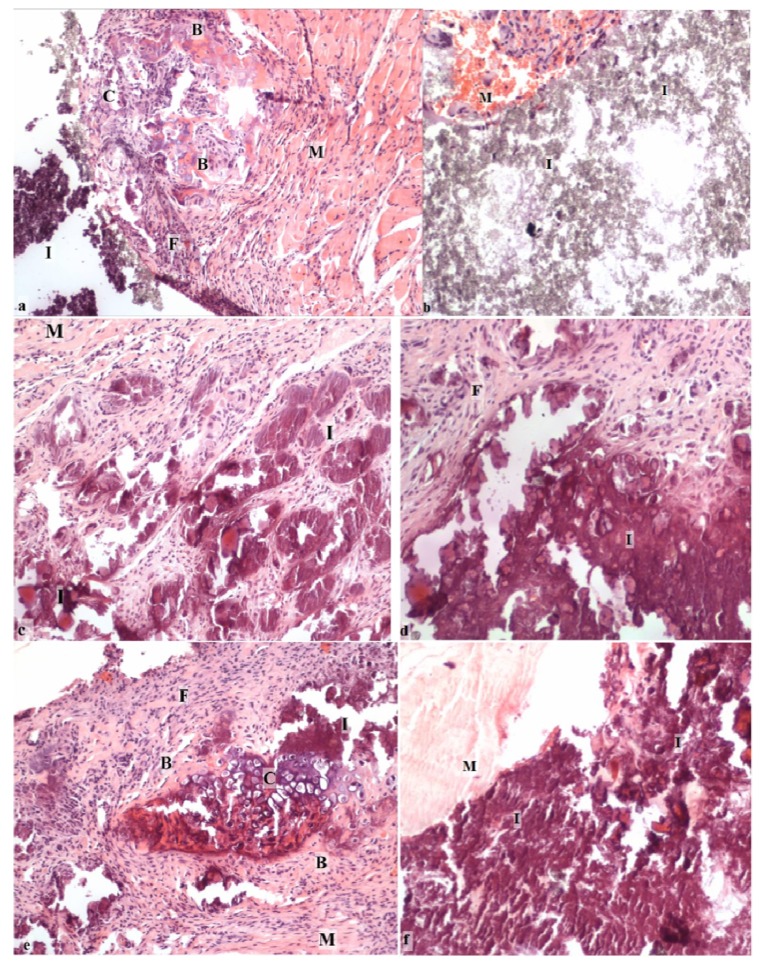
Histological examination showing the new bone formation and the implant response in the mouse pouch model using different carriers with the reindeer bone protein extract: (**a**) HAP/TCP/CS 30:60:10 active; (**b**) HAP/TCP/CS 30:60:10 control without bone protein extract; (**c**) CS hemihydrate active; (**d**) CS hemihydrate control; (**e**) CS dihydrate + stearic acid active; and (**f**) CS dihydrate + stearic acid control. C = calcified cartilage cells, B = bone, M = muscle, F = fibrotic tissue, and I = implant carrier. (Original magnification 10×).

For group a, the radiographic evaluation of the hydroxyapatite-tricalcium phosphate-calcium sulfate discs (HAP:TCP:CS 30:60:10) demonstrated some bone formation outside the active implant; however, the control implants remained intact (Table 1, Figure 3a). The measurement area was significantly higher for the active implants compared to the controls (*p <* 0.01). The harvesting analysis indicated that this new formation was bone-like. The histological evaluation demonstrated that endochondral bone formation occurred in the active sample and not in the control sample (Figure 4a, b).

For group b (TCP:CS 90:10), the radiography evaluation displayed some bone formation outside of the active implants; however, the control implants were nearly intact, and there were no statistically significant differences between the active implants and the controls (Table 1). Visual inspection during harvesting also indicated bone-like formations. The histological evaluation showed endochondral bone formation in the active sample and not in the control sample. 

For group c (Cem-Ostetic), the radiography analysis displayed no new visual bone formation; however, the measurement area was larger in the active group than in the control group (*p* < 0.01) (Table 1). Harvesting and histological analysis confirmed that no new bone was found in the samples. 

For group d (calcium sulfate hemihydrate discs), the radiography analysis revealed some new bone formation, and significant differences (*p* *<* 0.01) were apparent between the active and control groups (the control group had visibly resorbed) (Table 1, Figure 3b). However, the harvesting and histological analysis showed that no new bone was found in the samples (Figure 4c, d).

For group e (HAP/TCP/CS 60:30:10), the radiography evaluation showed some bone formation outside of the implant on the active side and some in the control implants (Table 1). Furthermore, the active group had a larger measurement area than the control group (*p* *=* 0.001). The harvesting analysis indicated that this new formation was bone-like. The histological evaluation revealed endochondral bone formation and mature cartilage cells in the active sample; however, none were found in the control sample. 

**Table 1:** Radiographic analysis of active implant containing the bone extract and control after 21-days follow-up (opalescent area in mm^2^). The percent increase compared to the control is shown.

Group	n	Active mm^2^ (SD)	Control mm^2^ (SD)	Increase %
(a) HAP/TCP/CS 30:60:10	8	34 (6.08)**^a^**	25 (3.14)	36
(b) TCP/CS 90:10	7	41 (12.22)	27 (1.41)	52
(c) Cem-Ostetic	7	76 (6.49)**^a, c^**	50 (6.04)	52
(d) CS hemihydrate	6	78 (13.47)**^a, c^**	44 (8.33)	77
(e) HAP/TCP/CS 60:30:10	8	46 (12.87)**^a, d^**	25 (2.77)	84
(f) CS dihydrate + stearic acid	7	97 (13.48)**^a, b^**	49 (13.38)	98

^a^*p* < 0.01 *vs*. control; ^b^*p* < 0.05 *vs*. other active groups; ^c^*p* < 0.01 *vs*. (a), (b) and (e); ^d^*p* < 0.01 *vs.* (a).

For group f **(**calcium sulfate dihydrate—stearic acid), the radiographic analysis and harvesting analysis revealed new bone formation in the active implant group (Table 1, Figure 3c). The difference between the active implants and the controls was statistically significant (*p <* 0.01). Also, histological analysis revealed clear bone formation and mature and calcified cartilage in the active sample (Figure 4e). No visual bone formation was apparent in the control sample (Figure 4f).

The comparison between all active groups revealed that group f had the largest measurement area, as determined by the radiographic analysis (*p <* 0.05). Furthermore, groups c and d had significantly larger areas than groups a, b and e (*p <* 0.01). There was also a statistically significant difference between active groups a and e (*p <* 0.05).

## 4. Discussion

The primary aim of this study was to find a suitable, inorganic, carrier candidate for reindeer bone protein extract. Six different candidates, including four different raw materials, were chosen to evaluate bone formation and implant resorption in the mouse pouch model with a three-week follow-up evaluation. In particular, calcium sulfate-stearic acid was an encouraging carrier candidate for the reindeer bone protein extract.

The reindeer bone protein extract has high bone formation activity, as seen in the bioactivity and previous tests (Figure 1); however, in a real bone healing situation, the extract cannot work without a scaffold system. Limitations of the carrier selection are set by the characteristics of the reindeer bone protein extract. The primary limitation is that the extract is not water-soluble. Thus, there are at least three different possibilities for implant preparation. The first is that the formulated bone extract suspension can be impregnated into a porous matrix (Figure 2a). The second method is to mold the extract and carrier together to form putty or compress them into the discs (Figure 2b), and in the third method, the carrier discs or granules are surface coated with the bone extract. Pure collagen has been tested as a carrier in some of our previous studies [18,19,21]. Lyophilized extract was mixed into water and then pipetted onto the collagen sponge; alternatively, the collagen sponge was soaked in water and then, with the extract, was bundled up to form an implant. The results of this method showed good bone formation in the pouch mice model and in the segmental defect model; however, it seems that collagen does not support the functionality of the bone forming proteins in the required time [24]. Therefore, an inorganic alternative would provide a better frame for the support of the bone healing effect of the extract. Previously, we have tested combinations of TCP, HAP and coral together with the extract and collagen sponge in the mouse model [17]. Furthermore, bioglass was found to be an acceptable carrier alternative as tested in the rat defect model [20].

This study was designed to find alternatives for carrier selection while considering the absorption of bone extract and the pore size characteristics of the carrier. With excipients, the lyophilized extract was absorbed into the pores of the TCP:CS 90:10 group, partially surface coated and partially absorbed into the HAP:TCP:CS 30:60:10 group. Furthermore, surface coating was used in the group of HAP/TCP/CS 60:30:10. The lyophilized reindeer bone protein extract was blended into the carrier material of the CS hemihydrate and the Cem-Ostetic groups and dry blended, without re-suspending the lyophilized extract, for the CS dihydrate-stearic acid group. Because the combination of the lyophilized formulation and the carrier was different for each study group, the distribution and availability of the extract was also different for each group; therefore, statistical comparisons between carrier groups are not valid. The native röntgengraphic method was used to determine the activity of implants in this study; however, this method cannot show bone formation inside of the remnants of the implant. The microtomography imaging method may give more detailed information on bone growth and carrier resorption in future studies. However, new bone formation was clearly seen in the histological analysis completed for this study.

All groups with extract performed better than the control groups without bone extract. The largest amount of bone formation was found in the groups that had the bone extract readily available, which indicates that the bone-forming factors are required at sufficient concentrations during the early stage. This was seen particularly in the HAP/TCP/CS 60:30:10 and CS dihydrate-stearic acid groups. In the TCP/CS 90:10, Cem-Ostetic putty and CS hemihydrate groups, differences between the active implants and the controls were observed, and the implants functioned as an implant with a bone-protein mixture coating. The smallest quantity of bone formation was found in the group HAP/TCP/CS 30:60:10, which indicates that the bone extract was absorbed deep into the scaffold during implant preparation, and the released quantity of bone proteins was too low to induce bone formation. These results support those from previous studies that showed that the formation of new bone depends on a ceramic content with a high HAP/TCP ratio and a high dose of bone proteins [5,9]. Furthermore, this study confirms that the presence of bioactive components reduced fibrous tissue formation and increased bone formation surrounding the inorganic scaffolds [9]. However, the quantity and availability of bone proteins should be in balance with bone healing and cascade formation [2]. 

The DBM products are comparable for reindeer bone protein extract [8,9]. The comparable amount of the commercially available DBM product had been also tested in the muscle pouch model but no sign of bone formation, either röntgengraphically or histologically, was seen within 21 days (data not shown). This indicates that proteins in the reindeer bone extract are more specific for inducing new bone, and bone formation capacity of extracted reindeer bone proteins is much better compared with the DBM. 

It is known that the presence of bone cells is essential for the degradation of calcium sulfate material. Ideally, bone formation and scaffold degradation follow one another until the defect area has been entirely replaced by new bone. If bone formation is not sufficient to supply mechanical strength, then the scaffold material should degrade slowly to prevent exposure of the support characteristics. This study also revealed that stearic acid positively affected the enhancement of bone ingrowth and formation in the environment of the calcium sulfate carrier. Stearic acid has been widely used as an excipient in tablet manufacturing because the addition of stearic acid decreases the viscosity of ceramic suspension while increasing the microstructural uniformity of particle packing [25]. Stearic acid is also used as part of plaster castings. Acid is sprayed on the surface of the casting mold that is parted after the casting. Then, stearic acid reacts with the calcium in the plaster to form a thin layer of calcium stearate, which functions as a release agent. Wright Medical Technology Inc. has used stearic acid as a tablet aid in their calcium sulfate products as Osteoset® and recorded good bone healing capacity, as found in previous work by the authors [15]. Thus, the conclusion is that calcium stearate not only has tablet-aiding properties, but also supports bone formation, similar to carboxymethylcellulose [26]. 

In conclusion, the greatest amount of bone formation occurred in the groups that had readily available bone extract near the surface of the implant. The combination of TCP or CS and stearic acid appeared to be the most ideal carrier alternative for reindeer bone extract. It was also suggested that the formulation of carrier materials as granules or in an injectable form would increase bone-formation efficacy. This hypothesis will be tested in further studies. 
